# Knowledge and Attitudes of two Latino Groups about Alzheimer Disease: a Qualitative Study

**DOI:** 10.1007/s10823-021-09432-0

**Published:** 2021-07-01

**Authors:** Laura Y. Cabrera, P. Kelly, I. E. Vega

**Affiliations:** 1 Department of Engineering Science and Mechanics, Center for Neural Engineering, College of Engineering, Pennsylvania State University, W-316 Millennium Science Complex, University Park, PA 16802 USA; 2 Rock Ethics Institute and Huck Institute of Life Sciences, Pennsylvania State University, University Park, PA USA; 3 College of Natural Science, Michigan State University, East Lansing, MI USA; 4 Department of Neurology, University of Michigan, Ann Arbor, MI USA; 5 Department of Translational Neuroscience, College of Human Medicine, Michigan State University, Grand Rapids, MI USA

**Keywords:** Alzheimer disease, Attitudes, Knowledge, Latinos

## Abstract

Clustering Latinos under a single group in Alzheimer Disease (AD) research, neglects, among other things cultural and environmental differences. To address this, we examine knowledge and attitudes about AD among two Latino groups. We held 5 focus groups and 2 interviews all in Spanish with Mexicans and Puerto Ricans between 40 and 60 years old living in the Grand Rapids area in Michigan. Using content analysis of the discussions, we identified themes related to knowledge, attitudes and concerns about AD and caregiving. A total of 20 Mexicans and 9 Puerto Ricans participated. *Improving knowledge and awareness*, *barriers* and *home-based family care* were important themes in both Latino groups. Puerto Rican groups raised more *concerns* about the disease, whereas *lack of knowledge* was a key theme among Mexican participants. The exploratory study is a first step in promoting research that is attentive to the commonalities and differences of Latino groups and in continuing efforts to enhance health literacy among these groups.

**Supplementary Information:**

The online version contains supplementary material available at 10.1007/s10823-021-09432-0.

## Introduction

Alzheimer’s disease (AD) is a neurodegenerative disease representing the most common cause of dementia in older adults (Alzheimer's Association, 2018). There are differences in the prevalence of the disease across ethnic groups in the United States, with some studies suggesting that Latinos are up to one-and-a-half times more likely to develop AD than non-Latino Whites depending upon the specific Latino ethnic group observed (for example, Mexican-Americans compared with Caribbean-Americans) (Alzheimer's Association, 2018; Mehta & Yeo, 2017).

Currently more than twelve percent of older adults within the Latino population are thought to have AD or a related dementia (NIA, 2018a), the highest proportion among different ethnic groups in the US (Mehta & Yeo, 2017). Moreover, as a group, the Latino community displays an earlier age on onset of AD in comparison to non-Latino whites (Mehta & Yeo, 2017) and have comorbidities known to contribute to disease onset such as a high prevalence of obesity, diabetes and metabolic syndrome (Heiss et al., 2014; Yracheta et al., 2015). There are important differences in AD risk factors among Latino groups, for example, Puerto Ricans have the highest prevalence of metabolic syndrome among Latino groups (37%), while Mexicans have higher proportions of overweight men and women compared to Puerto Ricans and Cubans (Heiss et al., 2014). There are also other important cultural and sociodemographic differences among Latino groups. For example, each group has its unique diets, with some Latino group diets been more affected than others via the acculturation process (Ayala et al., 2008). While Mexicans and Puerto Ricans are the two largest Latino groups in the US, the makeup of the Latino population varies widely across major metropolitan areas. Cubans are the group with the oldest median age (40 years) while Mexicans the group with the youngest (27 years) (Pew Research, 2019). Mexicans and Puerto Ricans have 12% and 19% of their population respectively who have attended college, while Venezuelans 55% (Pew Research, 2019). Latino’s economic conditions vary widely by origin group. The median household income of Puerto Ricans and Mexicans is very close (44,300 and 49,000 respectively), with other groups like Venezuelans presenting higher and others like Guatemalans, Dominicans and Hondurans lower. While only 8% of Puerto Ricans do not have health insurance, 20% of Mexicans do not have one. For Hondurans is even worst with more than one third of the Hondurans population in the US without health insurance (Pew Research, 2019).

Despite the higher prevalence and unique risk factors associated with Alzheimer’s disease in the Latino community, previous research shows that there are significant gaps in knowledge about the disease among the community, and perceptions about the disease are different when compared to other ethnic groups (Ayalon, 2013; Connell et al., 2009, 2017). There are also specific beliefs and values, which have been found to be more prevalent among Latinos (Ayalon, 2013; Azar et al., 2017; Gelman, 2010; Sayegh & Knight, 2013). For example, Ayalon’s study (Ayalon, 2013) found that people who identified as Latino, compare to white or black respondents, were significantly more likely to believe stress was a contributing factor to the development of AD and that exercise and mental activity would not play a role in disease prevention. Azar’s study (2017) also found that people who identified as Caribbean Hispanics, compared to white, were more likely to believe that AD was a natural part of aging, or that it was “God’s will.” This study also showed that this group was significantly less likely to be educated about AD and mental health in general. Regarding care practices, Mahoney et al. (2005) showed a high cultural aversion to nursing homes among Latinos family caregivers compared to African American and Chinese caregivers, with a high preference to caregiving for relatives at home and a generally higher sense of obligation to their families. Other studies also emphasized the role that cultural values like familism, the belief that the family supersedes the needs of the individual (Crist, 2002), and reciprocity have in the view that the elderly should be cared for by family (Apesoa-Varano et al., 2015; Aranda & Knight, 1997; Arévalo-Flechas et al., 2014; Gelman, 2010; Neary & Mahoney, 2005; Rote et al., 2019). A study by the Alzheimer Association reported that Latino and African American caregivers spend approximately 30 hours per week more time caregiving than non-Latino white caregivers (Alzheimer’s Association, 2015). Another study found that twenty-seven percent of Latino households provide informal caregiving to a friend or relative (Talamantes et al., 2006). Beliefs affecting interactions with the healthcare system among Latino groups, and other ethnic minority groups (including African Americans, Asian Americans), have been shown to be important barriers for seeking diagnosis and appropriate care of the disease (Chin et al., 2011; Janevic & Connell, 2001). Other studies have discussed lower levels of acculturation, culturally associated beliefs around the disease (such as memory loss being part of normal aging), and lack of knowledge about AD as perceived barriers to diagnosis and treatment of AD among Latinos (Sayegh & Knight, 2013; Gelman, 2010; Dilworth-Anderson et al., 2002). Together, perceptions rooted in cultural values and interactions with the healthcare system contribute to misconceptions about AD.

Although the difference in the Latino community’s knowledge and perceptions of AD compared to other ethnic groups is documented, there has not been significant research into differences in knowledge and attitudes towards the disease between different Latino ethnic groups. Other groups have argued for the need to account for differences across Latino subgroups in research (Aranda & Knight, 1997; Arévalo-Flechas et al., 2014).

Though Latino people are frequently grouped together based on language commonalities, this is an umbrella term for a highly geographically, culturally, racially, and genetically diverse group (Mehta & Yeo, 2017). Moreover, as mentioned above for those Latinos living in the United States there are also important differences regarding their historical experiences of immigration (if applicable) and acculturation to the United States (Arana, 2001), as well as differences in socioeconomic status, access to healthcare, English-language proficiency, and education.

This exploratory study focused on surveying similarities and differences in knowledge and perceptions of AD between Mexicans and Puerto Ricans. Identifying similarities and differences in the knowledge and perceptions of these groups can help target educational resources that are culturally sensitive to address information about the disease and care options.

## Methods

### Recruitment and Enrollment

Using a convenience sample, we conducted five focus groups (3 groups with Mexicans and 2 with Puerto Ricans) and two interviews about views and knowledge of AD with Mexicans and Puerto Ricans living in the Grand Rapids area in Michigan between February and June of 2019. The initial plan was to conduct three focus groups with Mexicans and three with Puerto Rican, however recruitment of a third group of Puerto Ricans proved to be very difficult, which motivated the recruitment of the only two confirmed participants from Puerto Rico not previously scheduled in a focus group for individual interviews. Following previous methodology (Cabrera et al., 2015), we used the same interview guide for each of the focus groups and the individual interviews. The area of Grand Rapids was chosen because the Latino community there spans several decades of history, forming throughout the mid-1900s and continuing today (Fernández, 2013), with a predominance of Mexicans but increasingly also from Puerto Ricans. As of the 2010 US Census, nearly 16% of people in the Grand Rapids area identified as Hispanic or Latino, being the city in Michigan with the second largest percentage of Hispanics and Latinos, many of whom identify as Mexican, Mexican–American, or Puerto Rican (United States Census Bureau, 2010). The study aimed to explore potential differences between two Latino groups regarding: (a) knowledge, attitudes, beliefs concerns around AD and its prevention, and resources available for its care; (b) perceptions and attitudes around care related to AD; (c) ethical issues related to Latinos and AD.

Participants were recruited with the support of the Michigan Center for Contextual Factors in Alzheimer’s Disease (MCCFAD) recruitment core, community organization newsletters, social media and hard copy postings. Respondents had to be between 40–60 years old or close to that range to be eligible to participate and were native Spanish speakers. We chose that age range to mitigate the possibility that participants might have already AD, while simultaneously ensuring an age group that was likely to be or have been caregivers for a parent or relative. Participants were screened through a telephone interview and excluded if they were not Puerto Ricans or Mexicans living in the Grand Rapids area. Eligible participants were scheduled for a focus group held in a community center in Grand Rapids or in a conference room at the Grand Rapids Research Center. We aimed to achieve a diversity of gender in each group and aimed to include between 6–8 people per group. Participants were compensated for their time and travel expenses with a modest reimbursement after completion of the focus group. Approval for the study was obtained from the Institutional Review Board of [Blinded for REVIEW] (IRB: 00,001,249) and participants gave written consent for their data to be used in the research.

### Focus Group and Interview Procedures

Before the start of the focus group, a research assistant (PK, Spanish as a second language) provided each participant with an informed consent form to read and the socio-demographic form to fill in. The facilitator (LC), a Spanish native speaker original from Mexico, then explained to participants the procedure of the focus group and addressed any questions. Participants were then invited to sign the informed consent form. Focus group discussions lasted approximately 1 hour, and were conducted in Spanish and facilitated by LC. All materials were made available in Spanish. Sessions were audio recorded. Field notes were taken by the research assistant to document verbal and nonverbal cues and other relevant information. For the interviews, these were conducted via zoom by the facilitator (LC) following similar procedures to those of the focus groups.

The facilitator followed a detailed, semi-structured focus group discussion guide that was informed by previous findings on cross-cultural views and knowledge about AD as well as upon ethical issues addressed in the available neuroethics literature published around AD (Johnson & Karlawish, 2015; Wikler et al., 2013). In addition, the focus group guide was also discussed and revised based on the input from a community advisory board, composed of Latino community leaders, to ensure consideration of cultural beliefs and context within the two Latino communities under study. An English-language version was developed first, and then translated into Spanish before implementation (Table 1). In consideration of cross-cultural and language differences, we utilized feedback from members of our Latino community advisory board to revise the focus guide. In addition, the translations from English to Spanish and vice versa were done by a member of the team (originally from Mexico) fluent in Spanish and English and revised both by an American student fluent in Spanish and another member of the team (originally from PR) fluent in Spanish and English. The Latino community advisory board approved the final version of the focus guide.

**Table 1:** Semi-structured focus group discussion guide

Disease: a. When you hear about Alzheimer's disease, what are the symptoms that you associate with the disease? b. What worries or fears do you have about Alzheimer's disease?
Diagnostic and Care: a. Have you heard of tests for the diagnosis of Alzheimer's? Which? What do you think about them? Do you have any concerns or fears about them? b. Have you heard of tests for Alzheimer's prognosis? Which? What do you think about them? Do you have any concerns or fears about them? c. What barriers / challenges do you think exist for the timely diagnosis of Alzheimer's? d. What do you think about the medical care associated with Alzheimer's? What challenges do you think exist (or have experienced) related to the medical care associated with Alzheimer's? e. What do you think about caring for family members with Alzheimer's at home?
Prevention: Do you know or have you heard of any way to prevent Alzheimer's or interventions that can help people with Alzheimer's?
Resources and Suggestions: a. What resources do you know in the community for the prevention and caregiving of Alzheimer’s disease? b. Do you have any suggestions on how to spread knowledge about Alzheimer’s disease c. Do you have any suggestions on how to address current challenges in the diagnosis or caregiving of Alzheimer’s disease? c. Is there anything else you would like to comment on your perceptions around Alzheimer's disease?

### Data Analysis

Recordings of the focus groups discussions and interviews were professionally transcribed and made ready for analysis in Microsoft Excel. A member of the research team corrected transcription errors and clarified inaudible speech and misattributed statements (PK).

Data was analyzed qualitatively using thematic content analysis by coding and organizing the data systematically (Boeije, 2002; Braun & Clarke, 2006). In the first phase of analysis PK and LC coded one transcript separately assigning codes to segments of the transcript texts; a draft codebook was created from discussing the different codes. A second transcript was coded using the draft codebook, and the coders discussed differences in coding, which lead to revisions of the codebook. Coded segments of data were then grouped into categories and themes that emerged from the data. Each category had a number of themes, and most themes had subthemes. The deliberations about the organization of the codebook were iterative until consensus was reached. After this, all focus groups transcripts were coded. Research team meetings were used to discuss questions about coding and accuracy of the codes. Inter-coder discrepancies were discussed until reaching coding consensus. For the Mexican focus groups, we reached data saturation, as no new themes emerged during data analysis, however for the Puerto Rican focus groups and interviews we cannot conclude with certainty that saturation was reached, even though no new themes emerged during data analysis of the compiled dataset for Puerto Rican transcripts.

We have structured the results section based on whether we found similarities or differences across groups for particular themes or not. We also identify themes that were dominant, a theme was categorized as dominant by its frequency and presence in the discussion in all focus groups from the same nationality, as well as its assertion by more than one participant within a given group. To complement this, statements that were regarded as typical or representative of a theme are presented. Following the current “gold standard” for translation technique we followed the double translation process (Arevalo-Flechas, 2009; Medrano et al., 2010).

## Results

We conducted five focus groups (3 groups with Mexicans and 2 with Puerto Ricans), each with 3 to 8 participants, as well as 2 interviews (*n* = 29 participants) (Table 2). All Mexican participants were foreign born and Puerto Ricans were originally from the island. Puerto Ricans participants typically had a better mastery of English compared to participants in the Mexican group.

**Table 2:** Participants demographics

	Mexican participants *N* = 20	Puerto Rican participants *N* = 9
Gender		
Female	12	8
Male	8	1
Age range (years)	39–60	40–56
Years of education (range)	0–16	8–20
Time living in grand rapids (range in years)	15–45	2–40

Another difference was that more Puerto Ricans had more direct experience with the disease, with two-thirds of participants having had experience with AD through a close family member (e.g. father, grandmother). However, only one of the participants reported being the primary caregiver for her father who was diagnosed very late in the disease progression with AD, the others discussed their experiences witnessing their parents or other extended family members being the primary caregivers of their grandparents.

The overarching themes of *improving knowledge and awareness* and *barriers* were prominent in all groups (≥ 10% of coded data for both groups). *Concerns* was a dominant theme for the Puerto Rican groups (13.5% of coded data), whereas *lack of knowledge* was a dominant theme among Mexican participants (18.8%).

### Points of Convergence: Priorities and Challenges

Discussion of priorities and challenges were key areas of convergence across both Latino groups. In terms of priorities, both Mexican (13.6%) and Puerto Rican focus groups (11.7%) discussed the importance of *improving knowledge and awareness*. Participants in both Latino groups mentioned a need for more education and resources around AD available to the Latino community, particularly resources in Spanish, as well as resources sensitive to cultural differences. They also recommended involvement of the community, suggesting involving organizations like churches and schools in disseminating information.

“*That this came to primary doctors…because you see with our diseases, we go to the doctor every month or every two months. We are always there, so they should have more information there with them*” (Mexican Focus Group 1, Female 5, translated by LC)

“*We should spread more information for the Latino people, that is available to more people, not only to those who have to go with a specialist, but to far away clinics, …in both languages, because what is happening is a lot of lack of information, for us, the Latino community*” (Mexican Focus Groups 3, Female 2, translated by LC)

*“[I] have seen things that have been translated into Spanish but that is not the same as saying, culturally appropriate for people [of those communities]”* (Puerto Rican Interview 1, Female 1, translate by LC)

Another important area of convergence was related to caregiving practices. We found that care by family at home is generally preferred in both groups, seen in many cases as part of someone’s duty after parents have taken care of them during childhood. These attitudes did not differ much from participants with and without AD experience. We have included in the participants quotes notes about their experience with AD.

*“I would not put my mom into an asylum, right?, because, you start thinking if they, with thousands of sacrifice, right? they help one grow, and no only one, it was, about ten, twelve [children]. They could with so many and why one is not going to be able with two or with one”* (Mexican Focus Group 1, Male 2, translated by LC; experience AD through an acquaintance)

*“Well, if the family can and [the person] can stay with the family, […] I think it would be the best for the person to help him not to be confused so much”* (Puerto Rican Interview 2, Female, translated by LC; experience AD with family member)

“*…let's say, we are several brothers and it is the mother or the father, and we work and we can help her and she stays with us and we take turns as a family, we help her because it is an affective commitment, than sending her to a like a ... nursing home or something like that*” (Mexican Focus Group 2, Male 2, translated by LC; have seen a movie about AD)

Nursing homes were mostly seen negatively (5/8 Mexican and 4/7 Puerto Rican participants commenting on the subject), as places where people do not receive the same attention and care as at home, or even as places where they are mistreated.

“*Because it has been seen on television sometimes that they are hit by the nurses or those who take care of them … For me, I say it's bad*” (Mexican Focus Group 1, Male 2, translated by LC; experience AD through an acquaintance)

*“We put our dad in a [nursing] home and it was terrible…”* (Puerto Rican Focus Group 1, Female 1, translated by LC; experience AD with family member)

“*all day is a schedule but it is not a schedule that has to do with the person but rather with the nursing home and the staff and, I felt very sorry to end this way when old and not have that control over one’s life*” (Puerto Rican Interview 2, Female, translated by LC experience AD with family member)

*“Because I feel that the love of the family, well we are going to do things to help that person, with love, with more love than a clinic…*.” Mexican Focus Group 2, Female 1, translated by LC; no AD experience)

Yet, there was acknowledgement in both Latino groups of circumstances (e.g., work responsibilities, worsening of symptoms, medications, safety) where care by a professional at home or in a nursing home might be better or necessary (3/8 Mexican and 3/7 Puerto Rican participants commenting on the subject).

*“My great-great-grandmother had everything, nephews, brothers, uncles, family, they could bathe her, as she should be bathed, feed as she wanted, give her the nutrition as she could, because there were so many people around her. Not here, society is very different, one has to work and go here, go there, it's not the same culture…”* (Puerto Rican Focus Group 2, Male 1, translated by LC; experience AD with family member)

“*It is a sacrifice, and it is difficult, and not everyone can do it, as such you cannot judge a person who has to leave a person in a place like a nursing home*” (Puerto Rican Interview 1, Female, translated by LC; experience AD with family member)

*“…it is very difficult for the children, to have to take one to that place, [...] I feel that if my daughters cannot take care of me, that it is better that they take me to that place because at least they will be giving me medicine even if it is to continue living and keep fighting, to finish, the journey of this life.”* (Mexican Focus Group 1, Female 2, translated by LC; experience AD through an acquaintance)

*“…a nursing home is better because in the nursing home they give you your medications and they put your shackle on, or your alarm so you do not get lost ... right? And well, for me, if I had that, I would like that they put me in a nursing home but that they would not forget me.”* (Mexican Focus Group 1, Female 5, translated by LC; no AD experience)

Another area of convergence was related to challenges, here *barriers* was a prominent theme discussed by both Latino groups (Mexicans 9.6%; Puerto Rican 10.2%). Barriers were discussed both in terms of diagnosis and treatment. Both groups mentioned issues regarding cost of care and lack of information. Puerto Ricans more frequently raised issues connected to *insurance* (5/9), whereas Mexicans issues connected to *the need for more specialists (6/20)*.

*“…, how much it will cost, and with insurance those are the barriers that one has to, in this system there is no reasonable way that one can go to a doctor and receive the treatment one wants”* (Puerto Rican Focus Group 2, Male 1, translated by LC)

*“But, I feel that the Latino community, and it has happened to me that I do think, this, how I am going to pay for those bills from the hospital, the big costs, that it implies, that you do those very thorough exams and that they really tell you that you have something serious.... [T]here is fear of how much we are going to pay*” (Mexican Focus Group 2, Female 1, translated by LC)

“[*B]ecause compare to what one earns, to the bills, one thinks ‘how many years will one take to pay that bill’*” (Mexican Focus Group 2, Male 1, translated by LC).

“*Yes, so many mental illnesses that one does not notice due to lack of information*” (Mexican Focus Group 2, Male 2, translated by LC)

*“I think that we haven’t given the importance needed to Alzheimer because there is not much information, or there was not much information before*” (Mexican Focus Group 2, Female 2, translated by LC)

*“I do not think there are many specialists in this. Specialists are needed*” (Mexican Focus Group 1, Male 1, translated by LC)

Another challenge mentioned by both Latino groups was *stigma*, mostly related to avoiding discussions on the topic or treating it as a joke.

*“[she quotes her mom saying] “yeah, and that’s a problem in our culture, we do need to talk about it”… because it’s a mental issue, mental illnesses don’t get looked at like diabetes, like heart, like lungs, it’s like “oh my gosh, it’s a mental disease, you’re crazy”* (Puerto Rican Focus Group 1, Female 3)

*“We talk about Alzheimer's but we talk about it as a joke ... I have many friends from different organizations and they say "oh no, it's that you have Alzheimer's, boy.*” (Puerto Rican Focus Group 2, Male 1, translated by LC)

Some participants also acknowledged that stigma seems to have decreased now compare to the past.

*“I think that nowadays, people are not the same as before, and at least we are a little more informed, but there is still stigma. For many people, out of fear or ignorance”* (Mexican Focus Group 2, Female 2, translated by LC)

### Other Points of Convergence: Views and Beliefs around Alzheimer Disease

In terms of general views and beliefs, both groups viewed the main symptom of AD to be *memory loss*, often mentioning problems with forgetting things. *Confusion* was another main symptom associated with AD among participants from both Latino groups, including confusion on how to use things or confusion stemming from becoming disoriented or not knowing who the people around them are. A few participants in both groups mentioned that the disease brought significant changes to the person, such as turning them aggressive or starting using bad words.

Participants from both Latino groups discussed AD as connected to *old age*.

“*I thought it was something you get when you are old*” (Puerto Rican Focus Group 1, Female 3, translated by LC)

*“Is normal because we know that older people lose their memory”* (Mexican Focus Group 3, Female 3, translated by LC)

Participants also acknowledged that in many cases people suffering with AD were considered to be *crazy*, although most of them emphasized that such view was not held by them.

*“We cataloged it that way, "he's crazy because of his age," but we do not know if that patient really has Alzheimer's or what's happening to him*” (Mexican Focus Group 2, Female 1 translated by LC)

“*But back then it was just like “she is crazy” [laughing] “leave her”, that’s what they would talk about*” (Puerto Rican Focus Group 1, Female 3, translated by LC)

### Points of Divergence: Concerns and Lack of Knowledge

One important area of divergence was related to the extent of the discussion and types of concerns raised by participants in both Latino groups. Puerto Ricans participants were more likely to discuss *concerns* (13.5%), in particular concerns about the e*ffects on loves ones* (7/9 participants) and the possibility of AD to *be hereditary* (5/9 participants).

*“Personally, I do not have family here, I do not have siblings, I do not have a mother, nothing. I have two children ... and if something happens to me, who will they be left with? Where will they be taken care of, will the government pick them up? Where are they going to end? you know? That is my fear "* (Puerto Rican Focus Group, Female 3, translated by LC)

“*Well yes one of the fears is that it is hereditary because in fact it has many people in my family, ah, grandparents on the mother and father side”* (Puerto Rican Interview, Female 1, translated by LC)

Another important difference we found in our study, was connected to a *lack of knowledge around the disease* being more common among the Mexican participants than among the Puerto Ricans participants (Mexican groups: 18.8%; Puerto Rican groups: 5.2%). Lack of knowledge was mostly manifested through several questions throughout the focus groups, in particular about the disease, disease progression and symptoms other than memory loss (7.4%), and there was also a lack of knowledge around diagnostic tools and causes.

For example, Mexican participants were more likely to not fully distinguish normal memory loss from more pathological forms of memory loss.

*“When something takes up all your attention but after you can’t remember where it is, and you are "think and think and where did you leave it" until you forget that thing and when you do not look for it, you find it.”* (Mexican Focus Group 1, Male 1, translated by LC)

Mexican participants were less likely to mention *preventive measures against AD*, whereas Puerto Ricans discussed a number of *preventative measures* including diet and exercise. Mexican participants were less likely to know of methods used to detect cognitive impairment associated with AD, whereas a majority of Puerto Ricans participants were aware of some methods (6/9 participants). Most Puerto Ricans held positive attitudes towards being diagnosed as possibly or probably having AD (8/9), mentioning that it would help towards better preparing them and their families for what is coming. Whereas, only a third of Mexican participants mentioned such positive attitude towards being diagnosed, but with a similar reasoning.

“*I would be afraid, but…[] I think I would like to know just for my family, maybe*” (Puerto Rican Focus Group 1, Female 1)

“…*perhaps, in some respects it would be the responsible thing to do, also because then one can make arrangements ... instead of leaving everything for the family to have to see, you know, to decide what they are going to do*” (Puerto Rican Interview, Female 1, translated by LC)

“*What I would be doing is preparing the family... Well, putting a plan so that we can help each other*” (Mexican Focus Group 3, Female 2, translated by LC)

## Discussion

In this cross-cultural qualitative study, we examined knowledge and attitudes about AD among the two largest Latino groups in the Grand Rapids area, Mexicans and Puerto Ricans. In particular, we focus on similarities and differences based on membership to a specific Latino group. We also explored key topics identified in previous literature discussing Latinos’ views and knowledge on the topic as a form of validation and elaboration.

There were more similarities than differences between these two groups. Our results reflect particular views and perceptions reported in previous studies comparing Latinos with other racial groups (Apesoa-Varano et al., 2015; Ayalon, 2013; Connell et al., 2017; Gelman, 2010; Mahoney et al., 2005; Rote et al., 2019). While major differences were not found between these two Latino groups, this could be a result of the low number of Puerto Rican participants. One possibility for the difference in number of participants is that, of the total number of Latinos living in the Grand Rapids area, 64% are Mexican compared to only 9% being Puerto Ricans. However, it is also possible that there are other cultural differences at stake as we tried several channels to contact Puerto Ricans, including via an electronic mailing list of Puerto Ricans, and via a Puerto Rican member from the Community advisory board directly helping us to reach the community, but it remained challenging to fully fill even a single focus group session. The difference in levels of research engagement between the Mexican and the Puerto Rican groups itself represents an important distinction worth studying.

Another important aspect to consider in the interpretation of our results is that having personal or professional experience with people with AD is likely to affect people’s knowledge and views about the disease. Two thirds of our Puerto Rican participants had close experience with AD, while only one Mexican participant had direct family experience (a grandmother). However, despite the direct experience with AD, no participant (in the Mexican group) was a primary caregiver. This might explain why *lack of knowledge* was a more dominant theme in the Mexican focus groups compared to the Puerto Rican ones, or that certain *concerns* (such as the effect on love ones or its heritability) were more frequently mentioned among Puerto Rican participants. Overall, for our sample, experience with AD does not seem to have an effect on attitudes towards caregiving, or ethical issues. Finally, education level is also an important aspect to keep in mind while interpreting the results, as the Puerto Rican groups on average had higher level of education compared to the Mexican focus groups, which again could be a reason for the predominance of *lack of knowledge* theme among the Mexican groups but not for the Puerto Rican ones. However, *lack of knowledge* among the Mexican groups could be a motivating factor to participate in activities related to AD. Thus, differences in motivation (e.g. biological, emotional, social, and cognitive forces) to seek information among Latino subgroups is another important area of study.

### Priorities and Challenges

In spite of previous findings showing the need for more education around AD among underrepresented ethnic groups (Gelman, 2010; Karlawish et al., 2011; Rote et al. 2019), our results highlight that this continues to be an urgent need. There is a strong desire from both Mexican and Puerto Rican participants to have more education and community involvement surrounding AD. To this end, several participants mentioned that it would be beneficial to host support groups or informational sessions in locally important locations like churches or schools. Similar to previous findings, our results show that given the prominent role that cultural and religious beliefs often play among minority communities, establishing ties with churches and other faith-based institutions is an important step for AD clinics (Dilworth-Anderson et al., 2008).

Previous studies have discussed various perceived barriers to diagnosis and treatment of AD among Latinos (Sayegh & Knight, 2013; Gelman, 2010; Dilworth-Anderson et al., 2002), including lower levels of acculturation, culturally associated beliefs and values (such as memory loss being part of normal aging), lack of accurate knowledge about AD, and health system barriers. Among our two groups, lack of knowledge, as well as cost and insurance continue to be important barriers, however contrasting with previous studies, our participants did not mention transportation, immigration status (Gelman, 2010) or stigmatization (Ayalon & Areán, 2004) as significant barriers to treatment or diagnosis.

It is unfortunately unsurprising that economic constrains (both costs of care and insurance coverage) continue to be important barriers among those two groups. Within the current health care system, people of color are less likely than whites to have health insurance and have historically had poor access to medical care (Sayegh & Knight, 2013; Fisher & Kalbaugh, 2011; NIA, 2018b). For Latinos living in the USA, disparities in the provision of health and human services are likely to be accentuated due to language as a barrier (Talamantes & Aranda, 2004). While language was not mentioned as a barrier per se, the majority of our participants, in particular those from Mexican origin, expressed a preference for having resources and information available in Spanish, in addition to English. Mexican participant’s preference for resources in Spanish may stem from the fact that English is not an official language as it is in Puerto Rico. This emphasizes previous knowledge on how culture and language might limit the extent to which these populations seek care or are aware of these opportunities (Gelman, 2010). Further research with larger samples could also help determine any differences in level of education and socioeconomic status regarding Latinos’ access and engagement with non-Spanish content regarding AD, and medical information more generally. Further investigation is also required to better understand the differences between Latino subgroups, taking in consideration healthcare seeking behavior. For example, due to their citizenship and political relationship with US, Puerto Ricans might be better equipped to navigate the healthcare system and seek resources, compared to other Latino subgroups.

### Attitudes around Caregiving Practices

Cultural values and beliefs can influence attitudes to care and caregiving practices (Dilworth-Anderson & Gibson, 2002). Compared to other racial groups, Latinos are known to have a stronger sense of duty to family (Gelman, 2010), undertaking caregiving as a family venture. This has also been referred in the literature as familism, perhaps one of the strongest and characteristic value found among Latino groups, and arguably one reason for older Latinos to be less likely than older non-Hispanic whites to live in long-term care facilities. Latino groups are also known for providing care without outside help longer than other groups (Aranda et al., 2003). In this regard, our results support the view that Latino people still prefer family-centered care at home for afflicted relatives, and this view was prevalent among both nationalities in this study. Cultural beliefs about duty to family and intergenerational reciprocity, as well as the value of “compromiso” (a sense of obligation in which children have a duty to help their parents in return for sacrifices made by their parents) were also part of their attitudes to family-centered care at home. Reciprocity in this regard seems to be rooted in childhood modeling of parents who took care of their own parents (Neary & Mahoney, 2005), but also on the idea that since their parents took care of them when they were young, then they are responsible for taking care of their parents when they are old and in need. A few participants also emphasized the importance of love as a driver to provide care to one own’s family. Given the strong cultural value of caring for one own’s family, it was not surprising to find mostly negative views about nursing homes, as they are seen as a breach of the family duty.

Previous research has found that Latino elders use nursing home services less often than Anglo elders (Crist, 2002) even when there are higher levels of need among them. Among the barriers for Latinos to use these services are duty to family, fear of discrimination, lack of knowledge about services, lack of sense of prevention, lack of health insurance, preference for traditional remedies and fear of neglect or abuse (Crist, 2002). In our sample, for example, some participants mentioned having heard of abuse in nursing homes which reinforces negative attitudes to these places and preference for care at home. However, compared to previous research, in which Latino participants had an unequivocally negative view of nursing home care (Mahoney et al., 2005), some participants were willing to consider placement in a nursing home when home care became impractical, such as in cases when responsibilities such as work prevented them from providing their loved one with full time care, or if their loved one began to exhibit severe aggression towards family members as the disease progressed. In this regard our findings align with other groups who have found that “Latinos do not unequivocally reject formal care options” (Apesoa-Varano et al., 2015; cf. Gelman, 2014; Rote et al., 2019).

We found acknowledgment that the culture in the United States was different than in Puerto Rico or Mexico, making it more difficult to provide care at home. As Latinos become more acculturated, it is possible that they may adopt the values of the wider culture and may be less likely to care for their relatives at home. Another important factor in changes in caregiving attitudes might be due to industrialization and social mobility (Crist, 2002). However, many participants struggled with adapting to the culture of the United States, where they do not have their extended family with them or where they cannot leave work in order to care for a family member. This struggle can make sending their loves ones to a nursing home a difficult, but sometimes necessary decision. In this regard, adherence to cultural values like familism can be reaffirming or stressful for Latino caregivers (Rote et al., 2019). While participants from both Latino groups shared culturally related beliefs about reciprocity, family obligations, and the primacy of home-based care, in comparison to previous studies about care practices among Latinos it seems that these tenets are weighted against practical factors, such as availability of family support and potential competing demands (work, health and care for other family members) (Apesoa-Varano et al., 2015; cf. Gelman, 2014; Rote et al., 2019), as well as considerations related to patient safety and financial constraints more than before.

There is a clear tension between cultural beliefs and the demands of individual’s circumstances, increasing the discrepancy between what is expected and what can realistically be provided (Crist, 2002). This finding is similar to previous findings among caregivers from various Latino groups (Neary & Mahoney, 2005), where participants expressed a firm commitment to family-based home care, while at the same time showed signs of adaptation to necessity given their particular context (Valle, 2014). These changing contexts for Latinos living in the US raise the issue of whether or not in the future there would be family members available to provide care for elders with chronic illnesses. Moreover, considering that Latinos are likely to experience greater psychological distress due to caregiving responsibilities (Talamantes & Aranda, 2004; Talamantes et al., 2006), it is important to better understand trends in caregiving and the factors affecting those.

### Knowledge and Beliefs

Knowledge of early signs and symptoms of the disease can lead to earlier detection of the illness, which can help improve the prognosis of the disease and can reduce the financial impact on families and communities impacted by AD (Leifer, 2003). Just as previous studies have shown (Gelman, 2010; Mukadam et al., 2011), we found that lack of knowledge about symptoms, prevention and resources around the disease continues to be a commonly mentioned barrier to diagnosis and care of AD. Many participants in both groups did not know the threshold at which symptoms become clinical, rather than part of normal cognitive decline due to age. This was particularly prevalent among participants without personal experience with the disease via a friend or family member. This might explain why Mexican participants tended to either not know other symptoms associated with AD beyond memory loss or confusion or to conflate unrelated symptoms such as normal lapses of memory with AD, as few of them had a relative or close friend afflicted by AD. Most Puerto Rican participants in this study had personal experience with AD which might have already exposed them to information and resources around it, making lack of knowledge a less common theme among the Puerto Rican group. Overall, participants from both Latino groups in our study are likely to believe that AD is a normal part of aging, which is a key difference found in research comparing Latinos as a group with other racial groups (Azar et al., 2017; Connell et al., 2017; Mahoney et al., 2005; Sayegh & Knight, 2013; Withers et al., 2019).

Contrary to previous research, our participants did not stress that AD was the result of living a difficult life or was caused by God (Ayalon, 2013; Dilworth-Anderson & Gibson, 2002), and while many did not distinguish between AD and other dementias, as indicated by the questions raised during the focus group, they all seem to acknowledge that it is a disease affecting the brain. *Stigma*, a significant theme in the normative and empirical literature on the topic (Ayalon, 2013; Mahoney et al., 2005; Sayegh & Knight, 2013), and a reportedly significant barrier to obtaining formal medical evaluation in groups with family-centered cultural values, was not a major theme identified in this study. Among the participants that touched on the issue there were several who thought stigma has been decreasing in past years. Several participants mentioned that the labelling of people with AD as simply “crazy” has seemed to fade away in recent years. Compared with the qualitative findings from Withers and colleagues survey (2019), no one in our sample believed that having a relative with AD was embarrassing, or expressed feelings of shame connected to the disease. This could be due to the fact that many participants in Wither’s study were based in rural Mexico compared to our population in Grand Rapids, who have already been living in a city for several years, which might have exposed them to more medical information. However, we did find that people avoid talking about the subject or that they use AD as a joke when someone forgets something. The use of humour to approach the disease might be a coping mechanism for something that is not well understood, and for which people fear, though some participants worried that it trivializes the severity of the illness.

### Attitudes towards Diagnosis

Previous studies explored attitudes towards diagnosis of Alzheimer’s in groups at-risk for autosomal dominant AD (Cabrera et al., 2015; Withers et al., 2019). Withers et al. (2019), found in their interviews with Mexican families that the main motivations to know own’s status was centered around preparing themselves and their families. Research of attitudes among the general public regarding early diagnostic testing for AD (Wikler et al., 2013) also showed that the majority of the public would be interested in obtaining a hypothetical early medical test for AD. In this regard, our results align with previous findings regarding both the willingness to know and the motivations for knowing, namely preparing their loved ones and prevention among Mexicans. In the case of many of our Puerto Rican participants, fear of the disease being heritable due to the familial history was also an important reason for why they would seek out diagnostic testing if it were readily available.

## Limitations

Findings represent a range of opinions from a small and selected sample of participants; thus, our findings cannot be generalizable. A major limitation is that due to the extreme difficulty in recruiting Puerto Rican participants for the focus groups, we ended up with a smaller number of participants,^1^ and had to replace the last Puerto Rican focus group with two interviews. The fact that focus groups aim to elicit views at the group level, whereas interviews do at the individual level, limits the type of inferences and comparisons of the combined data. The Hispanic Center of Western Michigan compiled multiple lists of Puerto Ricans who indicated interest in the study at recruiting events, but there was a very high rate of non-responses, and many people who were contacted either chose not to participate or were unable to attend the scheduled focus group sessions. This underrepresentation makes it difficult to solidly determine trends in beliefs and knowledge about the disease among Puerto Ricans, and future research into these groups’ attitudes towards the disease should try to understand recruitment differences among Latino subgroups and interview more Puerto Rican participants to better determine the predominant trends. In addition, considering that diagnostic and prognostic tests for AD are still not that accurate (with the use of standardized criteria, diagnosis is approximately 81% sensitive and 70% specific compared with the gold standard— pathology at autopsy) (Gaugler et al., 2013), that several of our participants had very low level of education, and that those questions could have been asked in a less leading way, answers to these questions add little value to the present study as such one should be cautious in the interpretation of our results. Another important point is that all Mexican Americans in the sample were born in Mexico so their perceptions of dementia may not represent the experiences and perceptions of Mexican Americans born in the US.

Finally, it is important to recognize that although the Mexican American community and the Puerto Rican community may have shared cultural ideals and beliefs, they are also heterogeneous with different socioeconomic and acculturation levels, resident status and beliefs.

## Conclusions

This exploratory study extends previous research on cross-cultural differences in Alzheimer disease. Overall, many of the beliefs and trends in knowledge fit with prior research into Latino perceptions of Alzheimer’s. There were more similarities than differences between Mexican and Puerto Rican participants’ knowledge and beliefs of AD and attitudes to care. This finding suggests that underlying implicit cultural values may override Latino interethnic differences. Yet crucial geographic differences that may impact acculturation processes should not be underestimated. For example, there might be important differences in communities living in highly dense Latino areas, or between those living in rural areas vs urban cities, all of these are important considerations affecting the negotiation of cultural values in different communities. This calls for further studies looking at potential interethnic differences and similitudes in and across different locations.

## Supplementary Information

Below is the link to the electronic supplementary material.

Supplementary file1 (DOCX 13 KB)

## Footnotes

1 We intended on finding at least 18 participants from each nationality, but we ultimately were only able to recruit 9 Puerto Ricans to participate.
