# Correction to: Knowledge and Attitudes of Two Latino Groups About Alzheimer Disease: A Qualitative Study

**DOI:** 10.1007/s10823-023-09478-2

**Published:** 2023-04-05

**Authors:** Laura Y. Cabrera, P. Kelly, I. E. Vega

**Affiliations:** 1 Department of Engineering Science and Mechanics, Center for Neural Engineering, College of Engineering, Pennsylvania State University, W-316 Millennium Science Complex, University Park, PA 16802 USA; 2 Rock Ethics Institute and Huck Institute of Life Sciences, Pennsylvania State University, University Park, PA USA; 3 College of Natural Science, Michigan State University, East Lansing, MI USA; 4 Department of Neurology, University of Michigan, Ann Arbor, MI USA; 5 Department of Translational Neuroscience, College of Human Medicine, Michigan State University, Grand Rapids, MI USA

**Correction to: Journal of Cross-Cultural Gerontology (2021) 36:265-284**

10.1007/s10823-021-09432-0

The author name “P. Kelly” was incorrectly listed as “K. Parker” in the original publication of this article. The original article has been corrected.

