# Supplementary material for: Knowledge and Attitudes of two Latino Groups about Alzheimer Disease: a Qualitative Study
Source: J Cross Cult Gerontol. 2021 Jul 1;36(3):265–84. doi: 10.1007/s10823-021-09432-0 (PMC8421275; doi:10.1007/s10823-021-09432-0)
Supplement: Supplementary file 1 — Supplementary file1 (DOCX 13 KB) [file 10823_2021_9432_MOESM1_ESM.docx]

**Appendix**

**Focus groups participant characteristics**

| **Focus group** | **# of participants** | **Age Range (years)** | **Years of Education Range** | **Time Living in Grand Rapids** **Range (years)** |
| --- | --- | --- | --- | --- |
| **Mexican FG 01** | 8 (6F/2M) | 42-59 | 4-16 | 16-45 |
| **Mexican FG02** | 5 (2F/3M) | 39-60 | 0-12 | 20-27 |
| **Mexican FG 03** | 7 (4F/ 3M) | 40-49 | 6-13 | 15-25 |
| **Puerto Rican FG 01** | 3 (3F) | 40-56 | 12-16 | 27-40 |
| **Puerto Rican FG 02** | 4 (3F/1M) | 40-56 | 8-20 | 2-34 |
| **Puerto Rican Interviews** | 2 (F) | 42-45 | 14 | 25-32 |
